# Platelet-Like Gold Nanostars for Cancer Therapy: The Ability to Treat Cancer and Evade Immune Reactions

**DOI:** 10.3389/fbioe.2020.00133

**Published:** 2020-02-25

**Authors:** Min Woo Kim, Gibok Lee, Takuro Niidome, Yoshihiro Komohara, Ruda Lee, Yong Il Park

**Affiliations:** ^1^International Research Organization for Advanced Science and Technology, Kumamoto University, Kumamoto, Japan; ^2^School of Chemical Engineering, Chonnam National University, Gwangju, South Korea; ^3^Faculty of Advanced Science and Technology, Kumamoto University, Kumamoto, Japan; ^4^Department of Cell Pathology, Graduate School of Medical Sciences, Kumamoto University, Kumamoto, Japan

**Keywords:** gold nanostars, blood cell membrane, biomimetic, immune escape, controlled release, targeted therapy

## Abstract

The cell membrane-coating strategy has opened new opportunities for the development of biomimetic and multifunctional drug delivery platforms. Recently, a variety of gold nanoparticles, which can combine with blood cell membranes, have been shown to provide an effective approach for cancer therapy. Meanwhile, this class of hybrid nanostructures can deceive the immunological system to exhibit synergistic therapeutic effects. Here, we synthesized red blood cell (RBC) and platelet membrane-coated gold nanostars containing curcumin (R/P-cGNS) and evaluated whether R/P-cGNS had improved anticancer efficacy. We also validated a controlled release profile under near-infrared irradiation for the ability to target melanoma cells and to have an immunomodulatory effect on macrophages. RBC membrane coating provided self-antigens; therefore, it could evade clearance by macrophages, while platelet membrane coating provided targetability to cancer cells. Additionally, the nutraceutical curcumin provided anticancer and anti-inflammatory effects. In conclusion, the results presented in this study demonstrated that R/P-cGNS can deliver drugs to the target region and enhance anticancer effects while avoiding macrophage phagocytosis. We believe that R/P-cGNS can be a new design of the cell-based hybrid system for effective cancer therapy.

## Introduction

Cell membranes are useful materials that are often employed in the drug delivery field. Inspired by their nature, many approaches to developing multifunctional drug carriers have been reported, which include so-called biomimetic drug delivery systems (Banskota et al., 2017). For instance, coating a variety of synthetic materials, including carbon, poly (lactic-co-glycolic acid) (PLGA), graphene, and gold nanoparticles with natural cell membranes such as those of cancer cells, stem cells, white blood cells, red blood cells (RBCs), and platelets (PLTs) have been widely used in the drug delivery field (Gao et al., 2017; Zhang et al., 2018a; Zhen et al., 2019). Cell membranes share properties with lipid-based nanoparticles and leverage these biological functions. Moreover, membrane-coated biomimetic drug delivery systems can load hydrophobic drugs into the phospholipid bilayer without any conjugation just as many lipid-based nanoparticles can. Hence, the membrane-coating strategy not only significantly increases the biocompatibility of nanoparticles but also improves their bioavailability (Fang et al., 2018).

Photothermal therapy (PTT) is a relatively non-invasive and safer method compared with conventional cancer treatment strategies due to its high accuracy (Okuno et al., 2013). Among PTT agents, gold nanoparticles are a promising candidate for cancer therapy because they are highly adjustable to surface modification, particle size, particle shape, and uniformity (Huang et al., 2007). All of these properties offer a variety of functions in biological systems and influence treatment efficacy. Gold nanoparticles are usually modified with polyethylene glycol (PEG) to enhance solubility, stability, and immune escape; however, there are many concerns about the PEGylation technique (Knop et al., 2010; Ishida and Kiwada, 2013; Zhang et al., 2017). PEGylation can activate the human complement system after repetitive administration, and the presence of anti-PEG antibodies may limit the therapeutic efficacy of gold nanoparticles. Instead of PEGylation, RBC membranes can offer a greater ability for immune escape (Piao et al., 2014), as RBC membrane proteins act as immunomodulatory antigens that help to avoid phagocytosis.

The exact mechanism of high temperature-mediated killing of tumor cells remains unclear. PTT can cause cellular necrosis at temperatures above 42°C, which can also be highly toxic to normal cells around the tumor region (Zhang et al., 2018b). Eventually, other approaches to broaden the application of gold nanoparticles are needed. For instance, the biomimetic strategy can combine PTT with currently available anticancer therapies such as chemotherapy to achieve improved therapeutic outcomes with increased safety (Hua et al., 2017). Biomimetic gold nanoparticles can enhance therapeutic efficacy under lower temperatures. Natural cell membranes are vulnerable to temperature changes, allowing the chemotherapeutics to be released from the membranes as the temperature is slightly increased (Roach et al., 2013; Kwon et al., 2015; Ebrahimi et al., 2018). This not only maximizes therapeutic effects but also prevents injury to adjacent healthy cells.

Considering its systemic toxicity and highly selective delivery, PTT agents are often used *via* intratumoral injection, and then subjected to irradiation by an external near-infrared irradiation (NIR) laser to generate heat for effective photothermal killing (Zhang et al., 2015). However, clinical applications of traditional PTT could be limited by the unpredictable tumoral nature; thus, a systemic approach is very meaningful. Well-fabricated gold nanoparticles can be localized to the desired tumor region *via* passive targeting, meaning the photothermal effect aids tumor penetration as well as killing the tumor (Zou et al., 2016). Nevertheless, gold nanoparticles without any functionalization are incapable of active targeting. To solve this problem, PLT membranes have been used to offer direct targeting (Jing et al., 2018). It has been demonstrated that PLT can recognize cancer cells as well as adhere to the damaged tumor vasculature or rapidly growing tumor vessels (Gay and Felding-Habermann, 2011; Goubran et al., 2014; Ortiz-Otero et al., 2018). Based on these rationales, in this study, we demonstrate the influence of versatile gold nanoparticles on therapeutic effects, cancer-targeting effects in melanomas, and immune escape ability from macrophages.

## Materials and Methods

### Preparation of Gold Nanostars (GNS)

Gold nanostars were prepared using a seed-mediated growth method (Yuan et al., 2012). All glassware and stirring bars were treated with aqua regia, rinsed with distilled water, and dried at 100°C. The seed gold nanoparticles were synthesized through citrate reduction of HAuCl_4_. Trisodium citrate dihydrate solution (1 wt%, 15 mL) was added to a reaction flask containing the boiling HAuCl_4_⋅3H_2_O solution (1 mM, 100 mL) under vigorous stirring. The reaction solution was heated for 15 min until the color turned from colorless to light red. Then, the solution was cooled and filtered using a 0.45 μM cellulose acetate membrane syringe filter. For GNS synthesis, the seed solution (30 μL) was added to the 20 mL glass vial containing the HAuCl_4_⋅3H_2_O (0.25 mM, 10 mL) and HCl (1 M, 20 μL) solutions at room temperature (RT) under stirring. Then, AgNO_3_ solution (3 mM, 100 μL) and ascorbic acid (100 mM, 50 μL) were quickly and simultaneously added to the reaction solution. This was stirred for 30 s until the color turned from light red to greenish-blue. The surface of GNS was functionalized with NH_2_-PEG-SH solution (4.5 mM, 200 μL). Finally, PEGylated GNS were centrifuged at 4,500 × *g* for 15 min, and then dispersed in distilled water.

### Preparation of Blood Cell Membrane-Coated GNS

Fresh mouse blood was collected from C57BL/6 mice by cardiac puncture for RBC and PLT isolation. RBCs and PLTs derived from this blood source were used in this study. To isolate RBCs and PLTs, the blood sample was centrifuged at 100 × *g* for 20 min at RT. Then, the blood was separated into PLT-rich plasma (PRP) supernatant and RBC sediment. The resulting PRP was centrifuged again at 100 × *g* for 20 min at RT to remove remaining blood cells. Then, PLTs were pelleted by centrifugation at 800 × *g* for 20 min at RT, and the pelleted PLTs were re-suspended in PBS (pH 7.4). PLT membranes were prepared by a repeated freeze-thaw method, and the resultant PLT membrane solution was centrifuged at 14,000 × *g* for 20 min. After washing three times, final PLT membrane samples were obtained and used to coat GNS.

Red blood cell ghosts (RBCs without hemoglobin) were prepared by treating RBCs in a hypotonic solution. RBC pellets obtained from the previous step were re-suspended in a 25% PBS solution and vigorously mixed by pipetting. RBCs were pelleted by centrifugation at 14,000 × *g* for 20 min at RT, re-suspended with PBS, and this process was repeated until all hemoglobin was released from the RBCs. RBC membranes were prepared by sonication, and the final RBC membrane samples in PBS were used to coat GNS.

Curcumin was dissolved in methanol and then diluted in a 1:100 ratio of a warm PBS solution. RBC and PLT membranes (RBC/PLT; 0.5:0.5 protein w/w ratio) were mixed with 500 μL of curcumin in PBS (0.1 mg/mL). The resultant mixture was sonicated in a water bath for 5 min and subsequently extruded 10 times through an 800 nm membrane filter using an Avanti extruder to encapsulate curcumin. Then, GNS were mixed with the blood cell membranes and kept for 30 min at RT. After incubation, the mixture was extruded 10 times through a 400 nm membrane filter to coat the GNS with blood cell membranes containing curcumin. The coated GNS were subsequently centrifuged at 4,000 × *g* for 15 min to remove excess blood cell membranes. These RBC and PLT membrane-coated GNS containing curcumin (R/P-cGNS) were then characterized and used for further experimentation.

### Physicochemical Characteristics of R/P-cGNS

The morphology of R/P-cGNS were characterized using a transmission electron microscope (JEOL-2100F, JEOL Ltd., Tokyo, Japan). An aliquot of R/P-cGNS solution was placed on a carbon-coated 400 mesh copper grid for 10 min. The solution was removed by gentle tapping, washed two times, negative-stained with uranyl acetate, and then dried. The prepared samples on the grid were observed with an electron microscope. The hydrodynamic size and zeta potentials of R/P-cGNS were measured using a Malvern Zetasizer (Nano ZS, Malvern Instruments Ltd., Malvern, United Kingdom). The absorption spectra of each component of R/P-cGNS were also measured in a microplate reader to verify successful membrane coating and curcumin encapsulation. The curcumin encapsulation rate and release profiles were measured for 1 h after sample incubation with 1% SDS to fully isolate curcumin from the membrane. Thereafter, the absorbance of the solution was measured using UV-vis spectrophotometer (Infinite M200 Pro, TECAN Group Ltd., Männedorf, Switzerland) at a wavelength of 424 nm (Supplementary Figure S1).

### Photothermal Properties the Prepared Materials

Thermographic images of water, blood cell membranes, GNS, and R/P-cGNS were captured to verify photothermal conversion. Each sample was exposed to an 808 nm NIR laser for 5 min, and the temperature changes were recorded at every time-point using a digital thermal image detector. Subsequently, NIR light-controlled drug release was also evaluated following 808 nm NIR laser irradiation. The curcumin release from R/P-cGNS (50 ppm) in a glass tube was monitored with and without irradiation. The concentration of released curcumin was calculated using a spectrophotometer at 424 nm.

### Flow Cytometry and Confocal Microscopy

Quantitative cellular uptake of different drug formulations (free curcumin, R-cGNS, P-cGNS, and R/P-cGNS) in B16-BL6 melanoma cells (ATCC, Manassas, VA, United States) was verified by flow cytometry (BD Accuri C6 Plus; BD Bioscience, San Diego, CA, United States) 3 h after treatment. Subsequently, the drug distribution pattern of curcumin in the cancer cells was observed by confocal fluorescence microscopy (BZ-8000; KEYENCE, Osaka, Japan) using the same conditions. Co-localization of R/P-cGNS and lysosome was confirmed by staining LysoTracker Green DND-26 (Invitrogen, Carlsbad, CA, United States) 3 h after treatment. The laser irradiation group was exposed to NIR laser for 5 min after 1 h of treatment, and then incubated for an additional 2 h.

### *In vitro* Cytotoxicity

B16-BL6 cells (5 × 10^3^, 100 μL) were incubated in 96-well plates at 37°C with 5% CO_2_ for 24 h. Then, media with the different drug formulations (curcumin, GNS, R-cGNS, P-cGNS, and R/P-cGNS) were added to the wells. Following drug addition, each condition was treated with and without NIR laser irradiation and further incubated for 72 h. Cellular viability was measured by the MTT assay at 595 nm. The cytotoxicity of GNS and free curcumin on B16-BL6 cells was also tested.

For colony formation assays, B16-BL6 cells (5 × 10^2^ cells, 200 μL) were prepared in tubes and treated with different drug formulations. Each condition was immediately exposed with and without NIR laser irradiation and seeded onto six-well plates. After 24 h, the cells were replaced with fresh medium and incubated for 10 days. After the 10-day culture, visible colonies were fixed with 100% methanol, washed with water, stained with 0.5% crystal violet in 25% methanol for 5 min, and washed with water until the excess dye was removed. After drying overnight, colony numbers were assessed visually. Pictures were taken using a digital camera.

### Gold Uptake by Macrophages

The immune escaping ability of various GNS formulations from phagocytosis was verified in the murine macrophage cell line, RAW264.7. To observe the nano-sized gold nanoparticles in the cytoplasm, a high concentration of GNS (100 ppm) was used to treat the macrophages, and their morphological changes and gold uptake were subsequently visualized with an optical microscope 4 h after incubation. The macrophages were fixed with 100% methanol and stained with 0.5% crystal violet in 25% methanol. Lipopolysaccharide (LPS) was used as a positive control.

To measure the actual uptake rate of gold atoms by macrophages, inductively coupled plasma-mass spectrometry (ICP-MS; iCAP 7000 Series, Thermo Fisher Scientific, Waltham, MA, United States) was used. Macrophages treated with each GNS sample 4 h after incubation were dissolved in aqua regia (a 3:1 mixture of hydrochloride and nitric acid), and the gold concentration was calculated compared with a gold standard curve.

### Immune Responses

RAW264.7 cells were seeded in 6-well plates at a density of 5 × 10^5^ cells/mL and incubated for 24 h. After incubation, the cells were treated with various concentrations of curcumin or various formulations of GNS for 2 h, and then stimulated with LPS (500 ng/mL) for an additional 6 h. A total of 1 mL of the cell culture supernatant was collected and levels of TNF-α, MCP-1, and IL-6 were detected using commercial mouse ELISA kits (430904, 432704, and 431304; BioLegend, San Diego, CA, United States) in accordance with the manufacturer’s protocols.

### Animal Study

The experiment was approved by the Institutional Animal Care and Use Committee (IACUC) of Kumamoto University (#A28-003) and conducted following protocols in accordance with the guidelines.

### Statistical Analysis

Data were analyzed by one-way or two-way analysis of variance (ANOVA) using Prism 7 (GraphPad Software, Inc., La Jolla, CA, United States). In all cases, the significance level was set at ^∗^*p* < 0.05, ^∗∗^*p* < 0.01, and ^∗∗∗^*p* < 0.001 or ^†^*p* < 0.05, and ^†††^*p* < 0.001 between the experimental groups; *n.s.* represents no significant difference.

## Results

### Preparation and Characterization of R/P-cGNS

**FIGURE 1:**
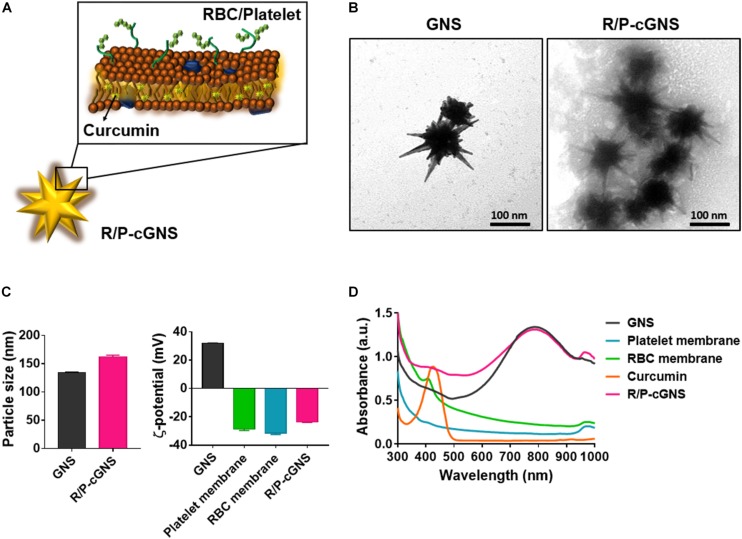
**(A)** Schematic illustration of R/P-cGNS. **(B)** Representative transmission electron microscopy images of GNS and R/P-cGNS showing the membrane coating structure (scale bar, 100 nm). **(C)** Particle size and surface charge of GNS and R/P-GNS, before and after coating with blood cell membranes. Error bars represent the mean ± SD (*n* = 3). **(D)** UV-vis absorption spectroscopy of GNS (black), PLT membrane (blue), RBC membrane (green), free curcumin (orange), and R/P-cGNS (pink).

To prepare R/P-cGNS, we first extracted cell membranes from RBCs and PLTs. Given the hydrophobicity profile of curcumin, it was encapsulated into the prepared blood cell membranes. Then the GNS were coated with these membranes *via* electrostatic interaction to fabricate R/P-cGNS containing curcumin as illustrated in Figure 1A and Supplementary Figure S2. The surface modification of the gold nanoparticles using each functional blood cell membrane brought different advantages such as immune escape ability and cancer-targeting. Therefore, we used the different GNS designs for ease of comparison. In the following study, GNS was used as the control. The functions of R-cGNS and P-cGNS were compared to R/P-cGNS; designs without curcumin were R-GNS, P-GNS, and R/P-GNS, respectively. TEM observations were used to characterize the morphology of GNS and R/P-cGNS. After coating with blood cell membranes, R/P-cGNS showed a uniform star shape with a membrane shell around GNS, indicating successful membrane coating (Figure 1B). SDS-PAGE protein analysis of P-cGNS, R-cGNS, and R/P-cGNS also proved that blood cell membranes were successfully coated onto GNS (Supplementary Figure S3). As shown in Figure 1C, the average hydrodynamic diameters of R/P-cGNS were slightly increased from 134.1 ± 1.2 nm in diameter to 162.1 ± 3.0 nm in diameter after membrane coating. The pattern of particle size distribution and the low polydispersity index (PDI) of 0.26 clearly verified no aggregation of particles (Supplementary Figure S4). Additionally, in response to the serum proteins in the 10% FBS solution, the particle size of R/P-cGNS remained constant, which implies that the particles possess colloidal stability (Supplementary Figure S5). The surface charge of R/P-cGNS (−23.7 ± 0.3 mV) decreased by over 50 mV compared with bare GNS (31.9 ± 0.2 mV) after membrane coating because the outer membrane surface exhibited a negative surface charge (Figure 1C). Additionally, the characteristic peaks of GNS (814 nm), blood cell membranes (300–700 nm, or 950–1000 nm), and curcumin (424 nm) in absorption spectra demonstrated that the membrane coating and drug encapsulation of R/R-cGNS had been successful (Figure 1D). The drug encapsulation efficiency and drug loading capacity were 71.6 ± 12.5% and 4.8 ± 0.8%, respectively (Supplementary Figure S6).

### Photothermal Properties and Drug Release of R/P-cGNS

**FIGURE 2:**
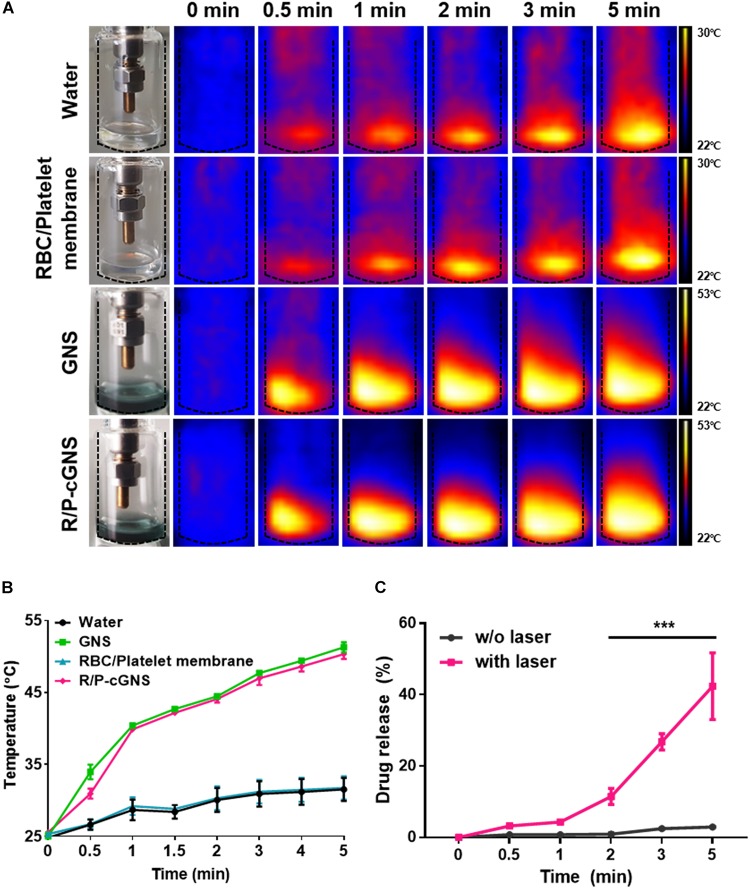
**(A)** Thermal digital images and **(B)** temperature increase curves of water (black), GNS (green), blood cell membranes (blue), and R/P-cGNS (pink) under NIR laser irradiation, as measured at different time points. **(C)** Curcumin release profiles from R/P-cGNS under NIR laser irradiation or without NIR laser irradiation (***p < 0.001, w/o laser vs. with laser). Error bars represent the mean ± SD (*n* = 3).

To investigate the effect of NIR laser irradiation on R/P-cGNS, temperature changes were measured using a digital thermal image detector (Figure 2A). There was no significant temperature change of water or RBC/platelet membranes over time, while the temperature curves of GNS and R/P-cGNS exhibited a rising pattern, which clearly indicated that GNS could generate hyperthermia under NIR irradiation and that membrane coating did not affect the photothermal effects of the GNS (Figure 2B). Within 2 min, the temperature increased to more than 42°C, which can induce photothermal therapeutic effects for cancer. The cumulative release profile of the drug suggested that hyperthermia can make blood cell membranes unstable, which then released the encapsulated curcumin. Driven by laser irradiation and the subsequent temperature increase, curcumin was rapidly released from R/P-cGNS, while no significant drug release was detected without laser irradiation (Figure 2C). These results indicated that R/P-cGNS could provide a controlled drug release under NIR laser irradiation, achieving both the expected photothermal and chemotherapeutic effects.

### Targeting Ability of R/P-cGNS

**FIGURE 3:**
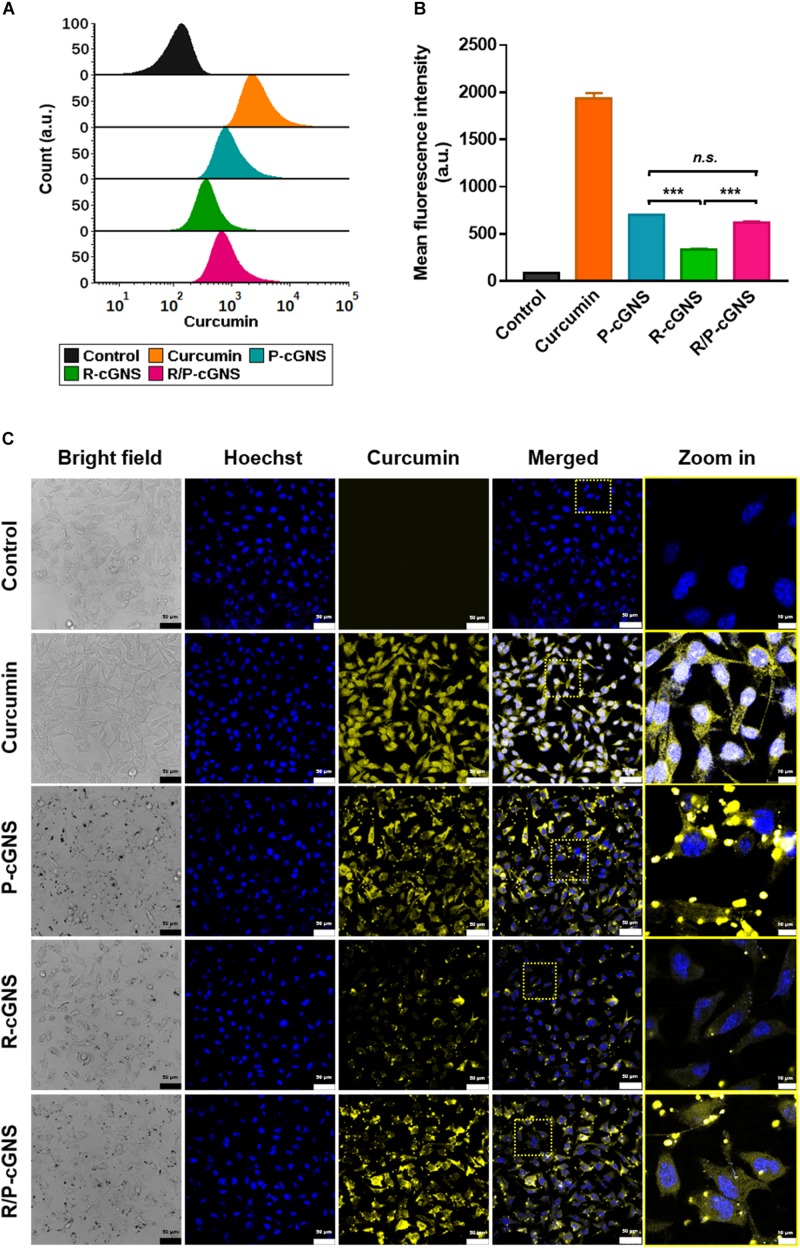
**(A)** Histogram plots of the fluorescence intensities showing cellular uptake of curcumin (orange), P-cGNS (blue), R-cGNS (green), and R/P-cGNS (pink) at a concentration of 25 μM curcumin. **(B)** The mean fluorescence intensities of different curcumin treatment groups with regard to the background level of untreated cells (black). Error bars represent the mean ± SD (*n* = 3) (***p < 0.001 vs. R-cGNS; *n.s.*, no significant difference, P-cGNS vs. R/P-cGNS). **(C)** Confocal images of different curcumin treatment groups in B16-BL6 cells.

B16-BL6 cells were incubated with an equal amount of curcumin, which was loaded in different GNS designs, and their drug delivery efficiencies on cancer cells were compared by flow cytometry (Figure 3A). The cells treated with curcumin exhibited a yellow color and we observed a distinct peak that was shifted to the right. A comparative analysis of curcumin uptake in B16-BL6 cells revealed that free curcumin showed the highest mean fluorescence intensity (MFI) value because it rapidly diffused into the cells *in vitro* (Figure 3B). GNS with PLT membrane represented a higher MFI value compared with that of GNS with a RBC membrane only, which was a result of the cancer-targeting ability. The MFI value of P-cGNS was slightly higher than that of R/P-cGNS, but there was no significant difference, which is assumed to be because PLT membrane contents for P-cGNS were twice as high as that of R/P-cGNS. These results indicated that R/P-cGNS has a targeting ability comparable to P-cGNS on cancer cells.

### Cellular Uptake and Distribution Profile of R/P-cGNS

To evaluate the cellular uptake of curcumin, the fluorescence properties after free curcumin, P-cGNS, R-cGNS, and R/P-cGNS treatment of B16-BL6 cells were examined by confocal microscopy (Figure 3C). Significant cellular uptake of curcumin was observed in the free curcumin-treated group, and it was evenly distributed in the cytoplasm; few R-cGNS could bind to cancer cells, and the fluorescent signal was weak. Conversely, when curcumin was delivered by P-cGNS or R/P-cGNS, the signal was relatively strong. Moreover, strong dot-shaped yellow fluorescence signals of curcumin were observed at the corresponding locations of GNS in bright field imaging, which demonstrates that the GNS was successfully attached to cancer cells (Supplementary Figure S7). Unlike free curcumin which is internalized into the cytoplasm through cytosolic diffusion, to clarify the intracellular uptake of R/P-cGNS, we tracked the location of R/P-cGNS by fluorescence labeling of the cellular compartment; R/P-cGNS was entrapped into endosome/lysosome 3 h after treatment, while curcumin could be successfully released from the vesicles by laser irradiation (Supplementary Figure S8).

### Cytotoxic Properties of R/P-cGNS

**FIGURE 4:**
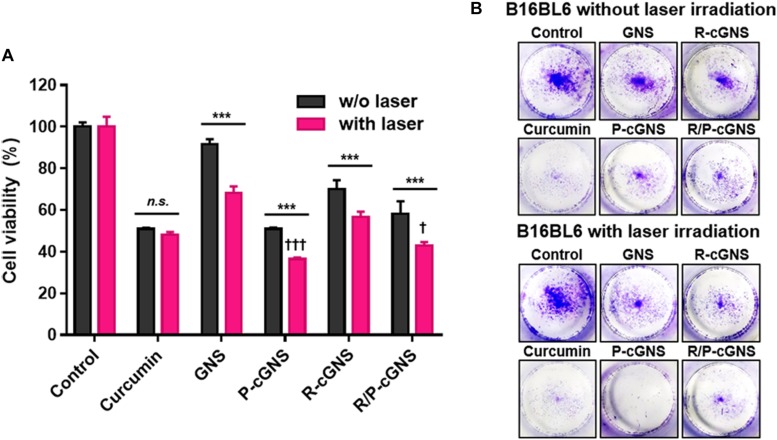
**(A)** Cell viability of B16-BL6 cells after incubation with different curcumin treatment groups under NIR laser irradiation or without NIR laser irradiation (*n.s.*, no significant difference and ***p < 0.001, w/o laser vs. with laser; ^†††^*p* < 0.001 and ^†^*p* < 0.05, vs. R-cGNS under NIR laser irradiation). Error bars represent the mean ± SD (*n* = 5). **(B)** Colony formation assays to determine the long-term effects of different curcumin treatment groups on cell proliferation.

Prior to proceeding to cytotoxicity test, the biocompatibility of GNS and the IC_50_ value of curcumin was evaluated. B16-BL6 cells were treated with bare GNS for 48 h, and the cytotoxicity of GNS was negligible even at the concentration of 30 ppm (Supplementary Figure S9). The IC_50_ value of curcumin was 32.6 μM as verified under the same condition (Supplementary Figure S10). Based on these results, the chemo/photothermal cytotoxicity potential of all GNS formulations without or with laser irradiation was evaluated by the MTT assay (Figure 4A). B16-BL6 cells were treated with curcumin at the IC_50_ concentration for 48 h. The free curcumin treatment group showed a considerable chemotherapeutic effect compared with the other groups, and NIR laser irradiation showed only a negligible therapeutic effect. Conversely, all groups irradiated by NIR laser after treatment of GNS formulations displayed significant cell death. The targetability of PLT membranes was further investigated and compared with R-cGNS. P-cGNS and R/P-cGNS showed higher cytotoxicity than R-cGNS. The cell viability was 36.4% for P-cGNS and 42.9% for R/P-cGNS, which was even lower than that of free curcumin (48.2%).

### Long-Term Therapeutic Effects of R/P-cGNS

Colony formation assays show only the metabolically active and viable cells after over 10-day incubation; thus, this is a valuable tool for overcoming the limitations of short-period evaluations using the MTT assay. Figure 4B shows the long-term therapeutic effects of GNS formulations and revealed the inhibition of colony formation of B16-BL6 cancer cells. A similar pattern to the MTT assay results was observed in the colony formation assays. Together these results indicated that R/P-cGNS could combine the photothermal effects of gold nanoparticles with the chemotherapeutic effects of curcumin under laser stimuli, leading to improved therapeutic effects in short- and long-term treatments.

### Immune Escaping Ability of R/P-cGNS

**FIGURE 5:**
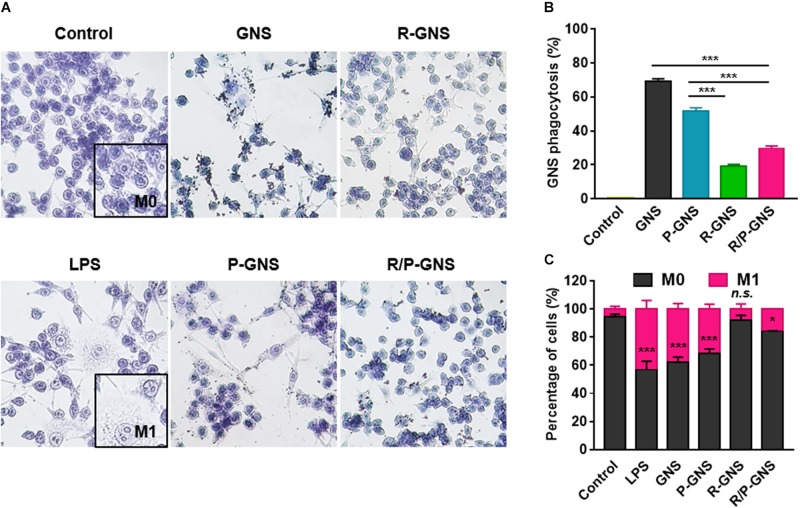
**(A)** Detection of blood cell membrane-coated GNS on RAW264.7 macrophages by optical microscopy and their morphological changes after 4-h exposure. **(B)** Quantitative analysis of GNS uptake using ICP mass spectrometry (***p < 0.001, vs. each group). **(C)** Analysis of the immunomodulatory activity of macrophages following treatment of immunostimulants such as GNS, R-GNS, P-GNS, R/P-GNS, and LPS (***p < 0.001, *p < 0.05, and *n.s.*, no significant difference vs. control). Error bars represent the mean ± SD (*n* = 3).

**FIGURE 6:**
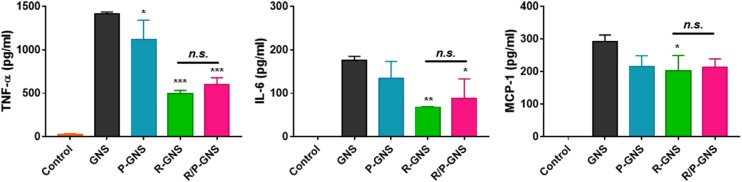
Quantification of the cellular production of cytokines in 264.7 macrophages following treatment with different GNS formulations (***p < 0.001, **p < 0.01, and *p < 0.05, vs. GNS; *n.s.*, no significant difference, R-GNS vs. R/P-GNS). Error bars represent the mean ± SD (*n* = 3).

Each of the GNS formulations was used to evaluate the phagocytic ability of macrophages. RAW264.7 murine macrophages were incubated with GNS formulations, and then intracellular gold nanoparticles were observed using light microscopy (Figure 5A). High concentrations of intracellular gold nanoparticles were observed as black spots in the cytoplasm of the cells. We found that macrophages treated with GNS accumulated more gold than blood cell membrane-coated GNS (Figure 5B). The surface coating of GNS with RBC membranes significantly inhibited phagocytosis (2.7-fold, ***p < 0.001) compared with GNS with PLT membranes. Additionally, R/P-cGNS (1.7-fold, ***p < 0.001) significantly improved the immune escaping ability to a similar extent as R-cGNS. These results indicated that R/P-cGNS had a comparable immune escaping ability to R-cGNS for macrophages. As shown in Figure 5A, control macrophages were round, whereas, after LPS stimulation, the macrophages changed into a spiky shape, suggesting that they were activated and ready to engulf foreign materials. This macrophage activation was also induced after GNS treatment. In general, activated macrophages produce cytokines such as TNF-α, IL-6, and MCP-1. The results of counting the number of activated macrophages and measuring the levels of pro-inflammatory cytokines clearly showed that R/P-cGNS, as well as R-cGNS, inhibited macrophage activation and balanced inflammatory responses (Figures 5C, 6).

## Discussion

There has been sufficient success in fabricating gold nanoparticles over the past decade that they can no longer be called a new attempt. However, combining the functionality of natural cell membranes from different origins has been insufficiently studied. In this work, we coated GNS with RBC and PLT membranes to simultaneously take advantage of immune evasion and cancer cell targeting abilities. The results of our experiments provide the underlying data for using RBC and PLT membranes together. We confirmed that RBC and PLT membrane-coated GNS could improve cancer targeting, alleviate immune responses, escape phagocytosis, and enhance anticancer effects. The use of gold nanoparticle coated with only a single membrane was slightly more effective than R/P-cGNS in some experiments; however, R/P-cGNS retained the functionality of both blood cell membranes. Considering the complex biological environment, R/P-cGNS might warrant further animal studies (Nel et al., 2009). The major immunomodulatory protein on the surface of blood cells is CD47, which is well known to give a “don’t eat me” signal (Burger et al., 2012). Although CD47 antigen, an important “self-recognition” protein, not only exists on RBCs, it is highly expressed on the surface of RBCs, helping to circulate RBCs in the bloodstream. The CD47 antigen enables cells to avoid phagocytosis by binding to the inhibitory receptor signal regulatory protein alpha (SIRPα) on macrophages, unlike PEGylation, which is a commonly used method to conceal the properties of nanoparticles by the hydration effect of PEG (Rao et al., 2015). According to the study by Hu et al. (2011) RBC membranes camouflaged nanoparticles have a prolonged circulation time in the blood compared with PEGylated nanoparticles by deceiving the immune system. In this study, we also found that RBC membrane coating can help to escape from macrophage phagocytosis; furthermore, the CD47-SIRPα interaction can directly regulate immune responses (Figures 5, 6). Additionally, curcumin can act as an anticancer drug as well as an anti-inflammatory drug; thus, it can induce immunosuppression even when R/P-cGNS are caught through macrophage phagocytosis (Supplementary Figure S11; Fadus et al., 2017). Over the past quarter-century, curcumin has attracted great attention due to its multitude of functions. Extensive clinical trials have addressed the efficacy of this versatile drug in cancer patients (Gupta et al., 2013). Despite considerable therapeutic efficacy, poor bioavailability has been a limitation to its use in humans. Since curcumin is sparingly soluble in aqueous solution at approximately 0.1 mg/mL, efforts are needed to improve the solubility of curcumin. According to Kurien et al. (2007), the solubility of curcumin in water could be maximized by dissolving first in methanol and then diluting with water or even using heat. Furthermore, the use of the cell membrane can improve the bioavailability of curcumin using lipid-based nanoparticles that capture hydrophobic drugs, and also allow intravenous injection (Kim et al., 2018). We also showed that curcumin has additional therapeutic benefits by using R/P-cGNS for cancer therapies including photothermal effects, controlled drug release at elevated temperature, and the targeting of cancer cells through PLTs. Recently, many trials have been implemented to study the use of the tumor-targeting properties of PLTs (Dehaini et al., 2017; Wang et al., 2019). PLTs primarily play an important role in hemostasis; the initial stage of hemostasis begins with the binding of activated platelets to the damaged vessels exposing von Willebrand factor (vWF), which prevents excessive bleeding (Yun et al., 2016). Thereafter, membrane proteins such as glycoprotein IIb/IIIa and glycoprotein Iba specifically bind to biomolecules like P-selectin, CD44, and vWF (Hu et al., 2015). These biomolecules are highly expressed in some types of cancer, including melanoma, hematoma, and osteosarcoma (Bauer et al., 2015). The interaction between the platelets and cancer cells has been reported to be related to thrombus formation and cancer metastasis (Mojiri et al., 2017). We found a therapeutic potential of R/P-cGNS on melanoma cells in particular; the melanoma cancer cell line B16-BL6 is reported to be effective in the treatment of curcumin and also to have a high expression level of vWF, which is the main target of PLTs. The results in Figure 3 reflect that R/P-cGNS have tumor-targeting ability because of the PLT membrane.

In this study, we prepared biomimetic GNS, R/P-cGNS, that were very delicately designed. Although R/P-cGNS have shown promising potential as a therapeutic strategy, it is still confronted with many difficulties in the biological environment. However, we are anticipating interesting results from star-shaped biomimetic gold nanoparticles, not sphere- or rod-shaped, from *in vivo* systems. This work was inspired by the features of PLTs, which normally circulate in the blood with a spherical shape in an inactive state and function only when they are activated and transform into a star shape (Jennings, 2009). We hypothesize that our results are related to recent findings regarding the correlation between the shape of the particles and their dynamic flow in the blood. It has already been reported that particle shape can affect particle bio-distribution and improve drug delivery (Tan et al., 2013). Further research will be needed in animal models or hemodynamic studies to explain these findings.

## Conclusion

To find some evidence for the potential of GNS in a biological environment, R/P-cGNS were investigated in this study. R/P-cGNS provide a controlled release profile, better targeting to cancer cells, immune escape, and enhanced therapeutic effects *in vitro*. The combination therapy of curcumin under NIR laser irradiation inhibited tumor growth. We also revealed that curcumin can provide anti-inflammatory effects on macrophages. Despite these advantages of R/P-cGNS, our system still requires further evaluation *in vivo*. As RBCs and PLTs are natural sources isolated from the blood, they might provide promising results for future preclinical trials. In conclusion, this study demonstrated that the newly developed blood cell membrane-coated GNS could be a good candidate for a biomimetic system that is ideal for cancer therapy.

## Data Availability Statement

The raw data supporting the conclusions of this article will be made available by the authors, without undue reservation, to any qualified researcher.

## Ethics Statement

The experiment was approved by the Institutional Animal Care and Use Committee (IACUC) of Kumamoto University (#A28-003) and conducted protocols in accordance with the guidelines.

## Author Contributions

MK, YP, and RL: conceptualization. MK, YK, and YP: methodology. MK and GL: investigation. MK and YK: validation. MK: formal analysis, visualization, data curation, and writing – original draft preparation. YP, GL, and TN: resources. MK, YK, YP, and RL: writing – review and editing. TN, YP, and RL: supervision.

## Conflict of Interest

The authors declare that the research was conducted in the absence of any commercial or financial relationships that could be construed as a potential conflict of interest.

## References

[B1] Banskota S. Yousefpour P. Chilkoti A. (2017). Cell-based biohybrid drug delivery systems: the best of the synthetic and natural worlds. *Macromol. Biosci.* 17:1600361. 10.1002/mabi.201600361 27925398

[B2] Bauer A. T. Suckau J. Frank K. Desch A. Goertz L. Wagner A. H. (2015). von Willebrand factor fibers promote cancer-associated platelet aggregation in malignant melanoma of mice and humans. *Blood* 125 3153–3163. 10.1182/blood-2014-08-595686 25977583 PMC4432010

[B3] Burger P. Hilarius-Stokman P. de Korte D. van den Berg T. K. van Bruggen R. (2012). CD47 functions as a molecular switch for erythrocyte phagocytosis. *Blood* 119 5512–5521. 10.1182/blood-2011-10-386805 22427202

[B4] Dehaini D. Wei X. Fang R. H. Masson S. Angsantikul P. Luk B. T. (2017). Erythrocyte-platelet hybrid membrane coating for enhanced nanoparticle functionalization. *Adv. Mater.* 29:1606209. 10.1002/adma.201606209 28199033 PMC5469720

[B5] Ebrahimi A. Csonka L. N. Alam M. A. (2018). Analyzing thermal stability of cell membrane of *Salmonella* using time-multiplexed impedance sensing. *Biophys. J.* 114 609–618. 10.1016/j.bpj.2017.10.032 29414707 PMC5985002

[B6] Fadus M. C. Lau C. Bikhchandani J. Lynch H. T. (2017). Curcumin: an age-old anti-inflammatory and anti-neoplastic agent. *J. Tradit. Complement. Med.* 7 339–346. 10.1016/j.jtcme.2016.08.002 28725630 PMC5506636

[B7] Fang R. H. Kroll A. V. Gao W. Zhang L. (2018). Cell membrane coating nanotechnology. *Adv. Mater.* 30:e1706759. 10.1002/adma.201706759 29582476 PMC5984176

[B8] Gao M. Liang C. Song X. Chen Q. Jin Q. Wang C. (2017). Erythrocyte-membrane-enveloped perfluorocarbon as nanoscale artificial red blood cells to relieve tumor hypoxia and enhance cancer radiotherapy. *Adv. Mater.* 29:1701429. 10.1002/adma.201701429 28722140

[B9] Gay L. J. Felding-Habermann B. (2011). Contribution of platelets to tumour metastasis. *Nat. Rev. Cancer* 11 123–134. 10.1038/nrc3004 21258396 PMC6894505

[B10] Goubran H. A. Stakiw J. Radosevic M. Burnouf T. (2014). Platelet-cancer interactions. *Semin. Thromb. Hemost.* 40 296–305. 10.1055/s-0034-1370767 24590421

[B11] Gupta S. C. Patchva S. Aggarwal B. B. (2013). Therapeutic roles of curcumin: lessons learned from clinical trials. *AAPS J.* 15 195–218. 10.1208/s12248-012-9432-8 23143785 PMC3535097

[B12] Hu C. M. Zhang L. Aryal S. Cheung C. Fang R. H. Zhang L. (2011). Erythrocyte membrane-camouflaged polymeric nanoparticles as a biomimetic delivery platform. *Proc. Natl. Acad. Sci. U.S.A.* 108 10980–10985. 10.1073/pnas.1106634108 21690347 PMC3131364

[B13] Hu Q. Sun W. Qian C. Wang C. Bomba H. N. Gu Z. (2015). Anticancer platelet-mimicking nanovehicles. *Adv. Mater.* 27 7043–7050. 10.1002/adma.201503323 26416431 PMC4998740

[B14] Hua H. Zhang N. Liu D. Song L. Liu T. Li S. (2017). Multifunctional gold nanorods and docetaxel-encapsulated liposomes for combined thermo- and chemotherapy. *Int. J. Nanomed.* 12 7869–7884. 10.2147/IJN.S143977 29123399 PMC5661837

[B15] Huang X. Jain P. K. El-Sayed I. H. El-Sayed M. A. (2007). Gold nanoparticles: interesting optical properties and recent applications in cancer diagnostics and therapy. *Nanomedicine* 2 681–693. 10.2217/17435889.2.5.681 17976030

[B16] Ishida T. Kiwada H. (2013). Anti-polyethyleneglycol antibody response to PEGylated substances. *Biol. Pharm. Bull.* 36 889–891. 10.1248/bpb.b13-00107 23727911

[B17] Jennings L. K. (2009). Mechanisms of platelet activation: need for new strategies to protect against platelet-mediated atherothrombosis. *Thromb. Haemost.* 102 248–257. 10.1160/TH09-03-0192 19652875

[B18] Jing L. Qu H. Wu D. Zhu C. Yang Y. Jin X. (2018). Platelet-camouflaged nanococktail: simultaneous inhibition of drug-resistant tumor growth and metastasis via a cancer cells and tumor vasculature dual-targeting strategy. *Theranostics* 8 2683–2695. 10.7150/thno.23654 29774068 PMC5957002

[B19] Kim M. W. Kwon S. H. Choi J. H. Lee A. (2018). A promising biocompatible platform: lipid-based and bio-inspired smart drug delivery systems for cancer therapy. *Int. J. Mol. Sci.* 19:E3859. 10.3390/ijms19123859 30518027 PMC6321581

[B20] Knop K. Hoogenboom R. Fischer D. Schubert U. S. (2010). Poly(ethylene glycol) in drug delivery: pros and cons as well as potential alternatives. *Angew. Chem. Int. Ed. Engl.* 49 6288–6308. 10.1002/anie.200902672 20648499

[B21] Kurien B. T. Singh A. Matsumoto H. Scofield R. H. (2007). Improving the solubility and pharmacological efficacy of curcumin by heat treatment. *Assay. Drug Dev. Technol.* 5 567–576. 10.1089/adt.2007.064 17767425

[B22] Kwon H. J. Byeon Y. Jeon H. N. Cho S. H. Han H. D. Shin B. C. (2015). Gold cluster-labeled thermosensitive liposmes enhance triggered drug release in the tumor microenvironment by a photothermal effect. *J. Control Release* 216 132–139. 10.1016/j.jconrel.2015.08.002 26247553

[B23] Mojiri A. Stoletov K. Carrillo M. A. Willetts L. Jain S. Godbout R. (2017). Functional assessment of von Willebrand factor expression by cancer cells of non-endothelial origin. *Oncotarget* 8 13015–13029. 10.18632/oncotarget.14273 28035064 PMC5355073

[B24] Nel A. E. Madler L. Velegol D. Xia T. Hoek E. M. Somasundaran P. (2009). Understanding biophysicochemical interactions at the nano-bio interface. *Nat. Mater.* 8 543–557. 10.1038/nmat2442 19525947

[B25] Okuno T. Kato S. Hatakeyama Y. Okajima J. Maruyama S. Sakamoto M. (2013). Photothermal therapy of tumors in lymph nodes using gold nanorods and near-infrared laser light. *J. Control Release* 172 879–884. 10.1016/j.jconrel.2013.10.014 24144919

[B26] Ortiz-Otero N. Mohamed Z. King M. R. (2018). Platelet-based drug delivery for cancer applications. *Adv. Exp. Med. Biol.* 1092 235–251. 10.1007/978-3-319-95294-9_12 30368756

[B27] Piao J. G. Wang L. Gao F. You Y. Z. Xiong Y. Yang L. (2014). Erythrocyte membrane is an alternative coating to polyethylene glycol for prolonging the circulation lifetime of gold nanocages for photothermal therapy. *ACS Nano* 8 10414–10425. 10.1021/nn503779d 25286086

[B28] Rao L. Bu L. L. Xu J. H. Cai B. Yu G. T. Yu X. (2015). Red blood cell membrane as a biomimetic nanocoating for prolonged circulation time and reduced accelerated blood clearance. *Small* 11 6225–6236. 10.1002/smll.201502388 26488923

[B29] Roach P. McGarvey D. J. Lees M. R. Hoskins C. (2013). Remotely triggered scaffolds for controlled release of pharmaceuticals. *Int. J. Mol. Sci.* 14 8585–8602. 10.3390/ijms14048585 23603890 PMC3645763

[B30] Tan J. Shah S. Thomas A. Ou-Yang H. D. Liu Y. (2013). The influence of size, shape and vessel geometry on nanoparticle distribution. *Microfluid Nanofluidics* 14 77–87. 10.1007/s10404-012-1024-5 23554583 PMC3611883

[B31] Wang H. Wu J. Williams G. R. Fan Q. Niu S. Wu J. (2019). Platelet-membrane-biomimetic nanoparticles for targeted antitumor drug delivery. *J. Nanobiotechnol.gy* 17:60. 10.1186/s12951-019-0494-y 31084622 PMC6513513

[B32] Yuan H. Khoury C. G. Hwang H. Wilson C. M. Grant G. A. Vo-Dinh T. (2012). Gold nanostars: surfactant-free synthesis, 3D modelling, and two-photon photoluminescence imaging. *Nanotechnology* 23:075102. 10.1088/0957-4484/23/7/075102 22260928 PMC3400343

[B33] Yun S. H. Sim E. H. Goh R. Y. Park J. I. Han J. Y. (2016). Platelet activation: the mechanisms and potential biomarkers. *Biomed. Res. Int.* 2016:9060143. 10.1155/2016/9060143 27403440 PMC4925965

[B34] Zhang X. Teodoro J. G. Nadeau J. L. (2015). Intratumoral gold-doxorubicin is effective in treating melanoma in mice. *Nanomedicine* 11 1365–1375. 10.1016/j.nano.2015.04.001 25888279

[B35] Zhang Y. Cai K. Li C. Guo Q. Chen Q. He X. (2018a). Macrophage-membrane-coated nanoparticles for tumor-targeted chemotherapy. *Nano Lett.* 18 1908–1915. 10.1021/acs.nanolett.7b05263 29473753 PMC7470025

[B36] Zhang Y. Zhan X. Xiong J. Peng S. Huang W. Joshi R. (2018b). Temperature-dependent cell death patterns induced by functionalized gold nanoparticle photothermal therapy in melanoma cells. *Sci. Rep.* 8:8720. 10.1038/s41598-018-26978-1 29880902 PMC5992202

[B37] Zhang Z. Qian H. Yang M. Li R. Hu J. Li L. (2017). Gambogic acid-loaded biomimetic nanoparticles in colorectal cancer treatment. *Int. J. Nanomed.* 12 1593–1605. 10.2147/IJN.S127256 28280328 PMC5339001

[B38] Zhen X. Cheng P. Pu K. (2019). Recent advances in cell membrane-camouflaged nanoparticles for cancer phototherapy. *Small* 15:e1804105. 10.1002/smll.201804105 30457701

[B39] Zou L. Wang H. He B. Zeng L. Tan T. Cao H. (2016). Current approaches of photothermal therapy in treating cancer metastasis with nanotherapeutics. *Theranostics* 6 762–772. 10.7150/thno.14988 27162548 PMC4860886

