# Supplementary material for: Platelet-Like Gold Nanostars for Cancer Therapy: The Ability to Treat Cancer and Evade Immune Reactions
Source: Front Bioeng Biotechnol. 2020 Feb 25;8:133. doi: 10.3389/fbioe.2020.00133 (PMC7051916; doi:10.3389/fbioe.2020.00133)
Supplement: Supplementary file 1 [file Data_Sheet_1.PDF]

# Platelet-like gold nanostars for cancer therapy: the ability to treat cancer and evade immune reactions

**Min Woo Kim[1†], Gibok Lee[2†], Takuro Niidome[3], Yoshihiro Komohara[4], Ruda Lee[1*] and Yong Il Park[2*]**

[1]International Research Organization for Advance Science and Technology (IROAST), Kumamoto University, Kumamoto, Japan

[2]School of Chemical Engineering, Chonnam National University, Gwangju, Republic of Korea

[3]Faculty of Advanced Science and Technology, Kumamoto University, Kumamoto, Japan

[4]Department of Cell Pathology, Graduate School of Medical Sciences, Kumamoto University, Kumamoto, Japan

**[†]These authors contributed equally to this work.**

**Running Title:** Platelet-like Gold Nanostars

*** Correspondence:**

Ruda Lee; aeju-lee@kumamoto-u.ac.jp

Yong Il Park; ypark@jnu.ac.kr

**Keywords: Gold nanostars, Blood cell membrane, Biomimetic, Immune escape, Controlled release, Targeted therapy**

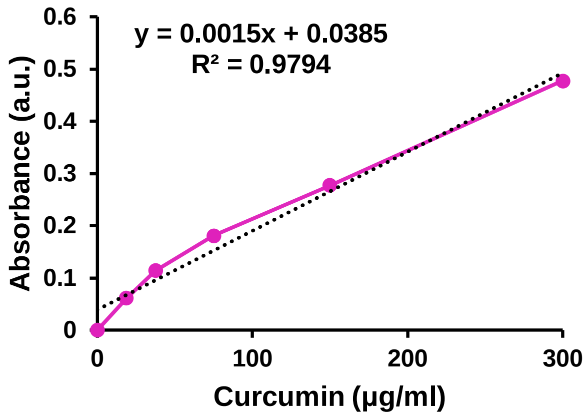


Figure S1. Standard curve of curcumin. Curcumin concentration was determined by measuring absorbance at 424 nm and comparing with a standard curve.

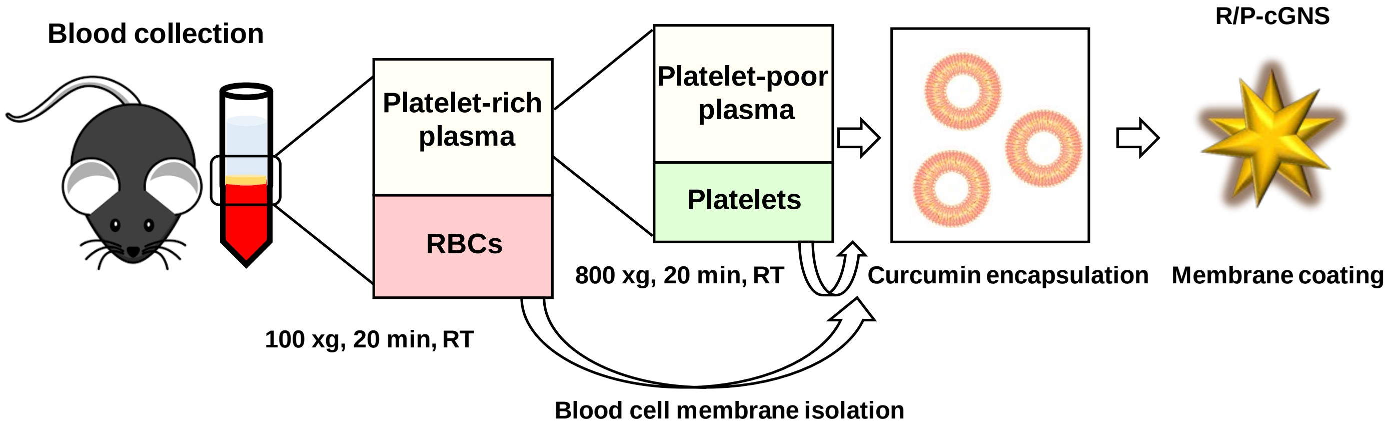


Figure S2. Schematic illustration of the preparation process of R/P-cGNS.

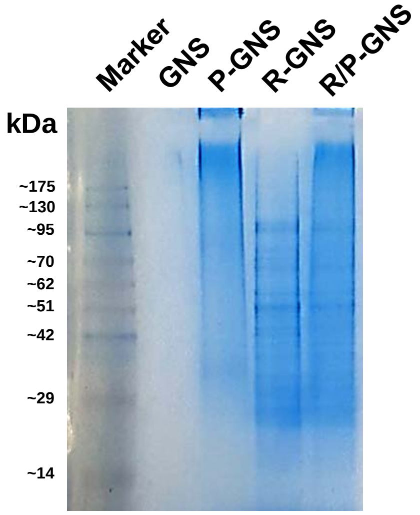


Figure S3. SDS-PAGE protein visualization of P-GNS, R-GNS, and R/P-GNS. The 10% polyacrylamide gel was stained with Coomassie blue. Protein molecular weight marker (PS11, GeneOn, Ludwigshafen, Germany).

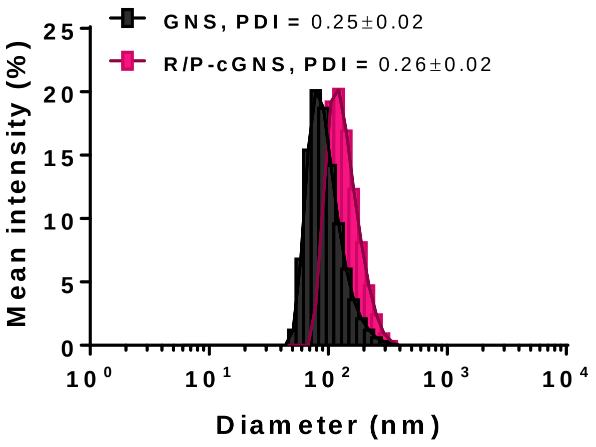


Figure S4. Size distribution patterns of GNS and R/P-cGNS.

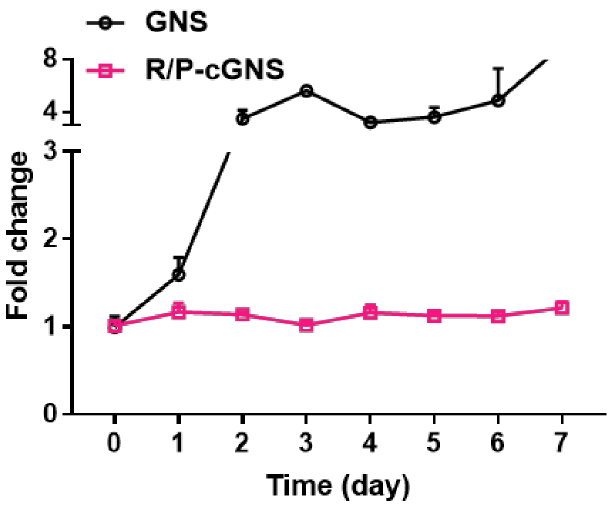


Figure S5. Stability analysis of GNS and R/P-cGNS in 10% FBS solution.

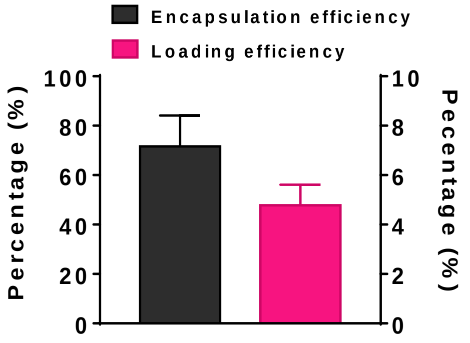


Figure S6. Encapsulation efficiency (E.E.) and loading efficiency (L.E.) of R/P-cGNS.

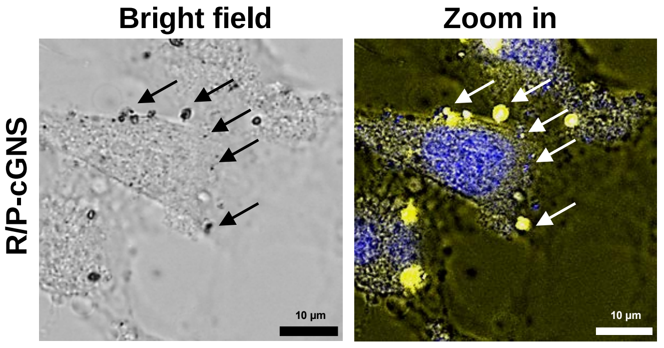


Figure S7. Comparison of bright filed and merged channels to detect R/P-cGNS on B16-BL6 cells. Arrows represent R/P-cGNS.

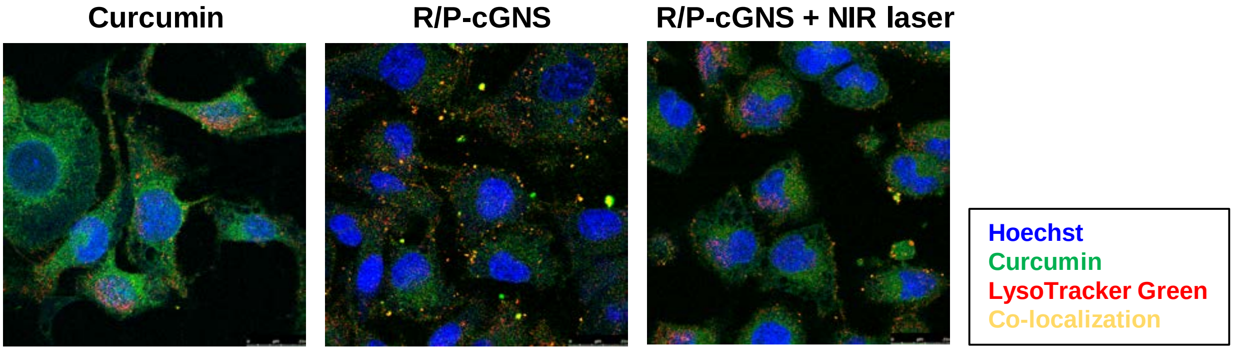


Figure S8. Intracellular localization of R/P-cGNS on B16-BL6 cells. The sample was incubated for 3 h. Curcumin was pseudo-colored as green for better visualization. Nucleus and lysosome were labeled with Hoechst 33342 (blue) and LysoTraker Green DND-26 (red; pseudocolored), respectively. Yellow fluorescent signals represent co-localization of lysosome and R/P-cGNS.

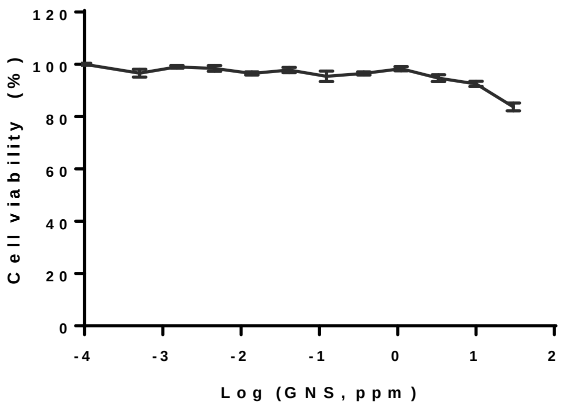


Figure S9. Cytotoxicity of bare gold nanostars on B16-BL6 melanoma cells.

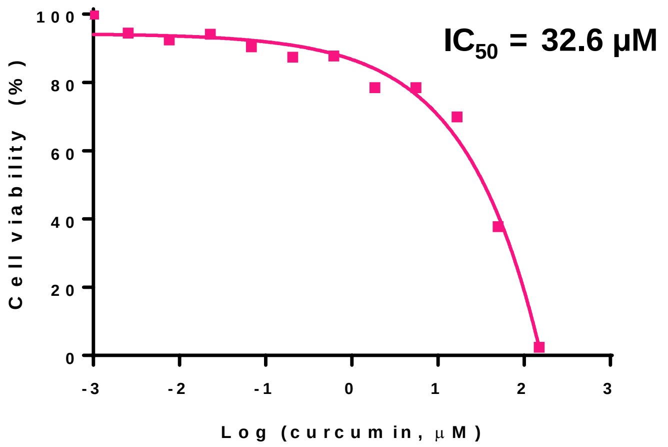


Figure S10. Evaluation of the IC50 value of curcumin on B16-BL6 melanoma cells.

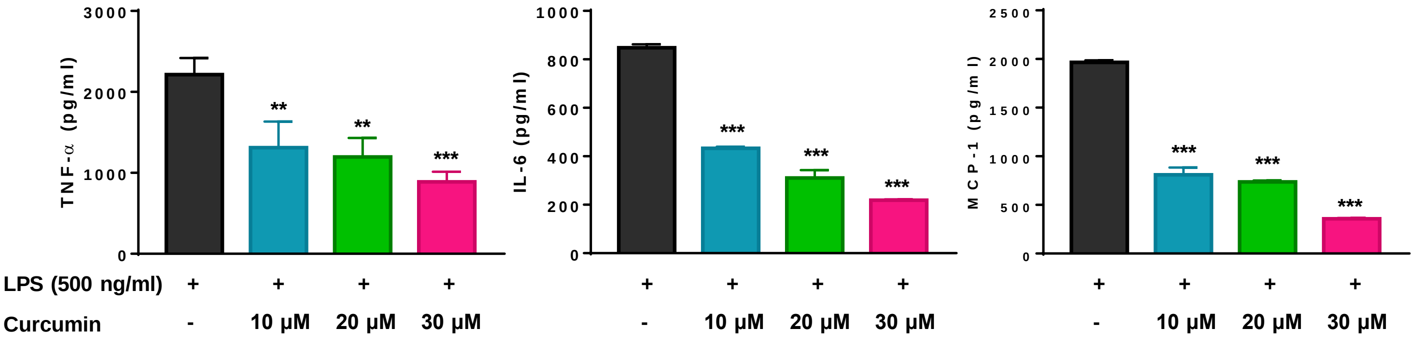


Figure S11. Immunosuppressive effects of curcumin on macrophages. The cells were incubated for 24 h. Curcumin treatment significantly decreased TNF-α, IL-6, and MCP-1 levels.
